# The Identification of Necroptosis-Related Subtypes, the Construction of a Prognostic Model, and the Characterization of the Tumor Microenvironment in Gliomas

**DOI:** 10.3389/fonc.2022.899443

**Published:** 2022-06-02

**Authors:** Yueyang Ba, Jiahao Su, Shuangqi Gao, Zhi Liao, Zhimin Wu, Chengan Cao, Chaofeng Liang, Jin Gong, Ying Guo

**Affiliations:** ^1^Department of Neurosurgery, The Third Affiliated Hospital, Sun Yat-sen University, Guangzhou, China; ^2^Department of Neurosurgery, Zhongshan City People’s Hospital, Zhongshan, China

**Keywords:** necroptosis, glioma, nomogram, tumor microenvironment, TMB

## Abstract

Necroptosis is a recently discovered form of cell death that plays a vital role in the progression of cancer, the spread of metastases, and the immunologic response to tumors. Due to the dual role of necrotic apoptotic processes in tumor pathogenesis and the heterogeneity of gliomas, the function of necroptosis in the glioma microenvironment is still poorly understood. We characterized the expression of necroptosis-related genes (NRGs) within glioma samples at both the genetic and transcriptional levels, identifying three distinct subtypes. Additionally, we constructed a risk score, which is capable of accurately predicting patient prognosis, correlates with tumor mutation burden (TMB), tumor stem cell index (CSC), immune checkpoints, and predicts tumor drug sensitivity. To facilitate its application in the clinic, we developed a nomogram and demonstrated that it predicts the prognosis of glioma patients with good accuracy and reliability using multiple datasets. We examined the function of necroptosis in the tumor microenvironment (TME) and the prognosis of gliomas, which may be useful for guiding individualized treatment plans for gliomas targeting necroptosis.

## Introduction

Among the many malignant tumors in the central nervous system, glioma is the most common, characterized by ease of recurrence and a high mortality rate (1). Diffuse gliomas are classified into 3 grades (WHO II, WHO III, WHO IV), and the higher the grade, the worse the prognosis (2).

Although the survival time of glioma patients has improved by using various treatments including surgery, radiotherapy, and chemotherapy, it is still far from expected (3, 4). With the development of molecular pathology, several molecular markers have been found to be relevant to the occurrence, progression, and prognosis of gliomas. These markers include methylation of the MGMT promoter, IDH mutations, and co-deletion of chromosome 1p/19q (5, 6). However, due to the high heterogeneity of gliomas, these markers are currently not fully adequate for predicting prognosis and guiding individualized therapy. Thus, it is imperative to construct reliable new prognostic models to predict the microenvironment of the tumor and to guide individualized therapies.

Necroptosis, a cystathionine-independent programmed death, is regulated mainly by receptor-interacting protein [RIP] kinase 3 (RIPK3), RIPK1, and mixed-lineage kinase structural domain-like pseudokinase (MLKL) (7, 8). As a new form of necrotic cell death, necrotrophic apoptosis is indispensable for the biological processes of cancer development, progression, metastasis and prognosis, and immune surveillance (9–14). In tumors, it has dual functions; its key mediators promote tumorigenesis and progression (15, 16); and it also prevents tumor development when apoptosis is compromised (17, 18). Targeting necrotic apoptosis is a promising immunotherapeutic approach for eliminating tumor cells when tumors become apoptosis-resistant. A variety of drugs and chemotherapeutic agents that have been approved for clinical trials are selective necroptosis inducers for specific tumors (11). Due to the high heterogeneity of gliomas, the impact of necroptosis on their prognosis and immunotherapy is not well elucidated. Because of technical limitations, most research has focused on individual drug or mediator targets, whereas immunotherapy and prognosis are the results of all relevant genes working in concert. Therefore, there is an urgent need to comprehensively understand the impact of necroptosis markers on the TME, immunotherapy response, and prognosis of glioma. However, the role played by necroptosis-related genes in the development and prognosis of glioma has not been effectively elucidated by current studies.

In this research, we comprehensively assessed the immune landscape of necroptosis-related genes (NRGs) in glioma patients. We explored the effect of NRGs on the TME and immunotherapy of gliomas. We then constructed a risk score capable of accurately predicting tumor immune status, prognosis, and sensitivity to chemotherapy. The final risk score was combined with clinical data to construct a nomogram that provides guidance for the clinical application.

## Materials and Methods

### Datasets

**Figure 1:**
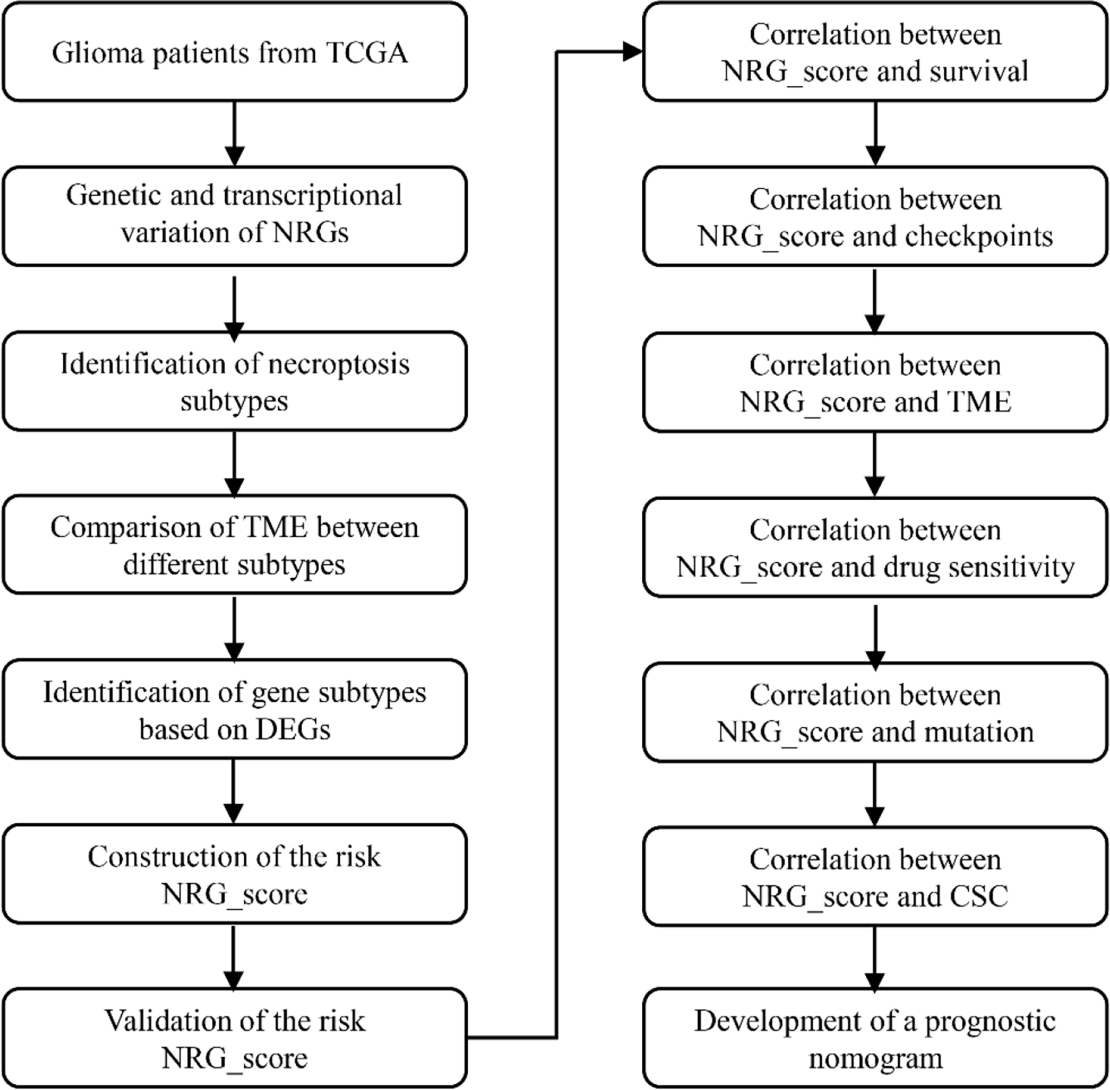
The entire analytical process of the study.

In **Figure 1**, we present an overview of the analysis of this research. The Cancer Genome Atlas (TCGA) database was searched on January 1, 2022, to obtain the RNA transcriptomic datasets (HTSeq- per kilobase million (FPKM) and HTSeq-Counts) and associated clinical information for the patient’s 5 normal brain tissue samples and 698 glioma samples. RNA transcriptome data (FPKM and counts data) from normal brains were obtained from the GTEx database (http://xena.ucsc.edu/) on January 1, 2022. RNA transcriptome data and corresponding clinical data were collected from the Chinese Glioma Genome Atlas (CCGA) database for 693 and 325 patients with glioma (12).

The GTEx and TCGA datasets were merged to remove batch effects by the “Combat” algorithm, and the CGGA dataset was merged in the same way. Fragments We converted the values as transcripts per kilobase million (TPM) from FPKM. The Counts value matrix was used only to identify differential expression, while the TPM matrix was used for other analysis. Patients with missing overall survival values and duplicate samples were excluded when constructing models for prognostic analysis. The TCGA set was randomly assigned to the training and test groups by Perl and caret R packages in a ratio of 2:1. For validation of the prognostic model, the Rembrandt and CGGA datasets were utilized as the external validation datasets. The clinical data of these datasets are available in **Supplementary Tables 1, 2**. Since TCGA, Rembrandt, and CGGA databases are open access data and publicly available, this research did not need ethical approval. The copy number variation (CNV) and somatic datasets of glioma were also collected from the Xena and TCGA, respectively. Data analysis was performed using Perl (5.34.0) and R (version 4.1.0).

### Copy Number, Mutation Analysis, and Differential Expression Analysis of NRGs

From the necroptosis gene set M24779.gmt of the MSigDB (http://www.broad.mit.edu/gsea/msigdb/) and previous reports on necroptosis (19), we ended up with 67 necroptosis-associated genes (**Supplementary Table 3**). The Perl and R software were applied for CNV analysis, the ‘RCircos’ package was applied to show the distribution of NRGs alterations in chromosomes, and the ‘maftools’ were used to map the oncoplot of gene mutation. The ‘limma’ and ‘reshape2’ packages were used to identify differences in NRGs expression in normal brain tissue and gliomas. We further performed protein-protein interaction (PPI) networks for 67 NRGs using the STRING (https://cn.string-db.org/) and the interaction score was set to the highest confidence (0.9). The top 50 differentially expressed NRGs among them were visualized using ggplot2 for correlation analysis.

### Consensus Clustering Analysis of NRGs

Prognostic risk network plots were drawn using the ‘RColorBrewer’, ‘psych’, ‘igraph’, and ‘reshape2’ packages. Based on the expression levels of NRGs, patients with glioma were classified into distinct NRGclusters using the R package “ConsensusClusterPlus”.

### Clinical Features and Prognosis of Glioma Based on Molecular Subtypes

A correlation analysis was applied among the differences in molecular subtypes, as determined by consistent clustering, and different clinicopathological characteristics and prognosis. Clinical and pathological characteristics of the patients included survival time, survival status, gender, age, WHO grade, the status of radiation therapy or chemotherapy, the presence of mutations for IDH, 1p/19q codeletion, and MGMT promoter phosphorylation. Using the “survminer” and “survival” R packages, we examined prognostic differences of different subtypes based on Kaplan-Meier curves.

### TME, PD-L1, and PD-1 in Different Molecular Subtypes

Our evaluation of each patient’s immune and stromal component was performed utilizing the ESTIMATE. To evaluate the proportions of distinct immune cell types, the CIBERSORT algorithm was applied (20). By using “ssGSEA” in the R package “GSVA” (21), we quantified the levels of cellular infiltration of the immune system. Further analysis was conducted on the expression of PD-1 and PD-L1 among the subtypes.

### Identification and Functional Enrichment of DEGs

Functional enrichment analysis of DEGs between distinct subtypes was conducted with the “clusterprofiler” package (adjusted p-value < 0.05). The clusterprofiler is a popular machine learning algorithm, which was extensively utilized in medical studies (22–27).

### Construction of the NRG_Score

NRG_score was constructed to evaluate the necroptosis of tumors. A univariate Cox regression analysis was applied on these prognostic DEGs among NRGclusters. Following this, patients were classified into four geneClusters using the consensus clustering analysis. The consensus clustering is a popular bioinformatics algorithm, which was extensively utilized in cancer-related studies (28–32).

Patients with missing overall survival values and duplicate samples were excluded. Patients with glioma from TCGA datasets were randomly classified into the training and test groups by the Perl and Caret R packages in a ratio of 2:1. For the training set, the NRG_score was figured out using Lasso Cox and multivariate Cox regression analysis. In calculating the NRG_score, the following formula was used:


NRG_score=∑(Expi∗Coefi)

Expi and Coefi of this equation correspond to the expression and risk coefficients, respectively. A median risk score was used for dividing patients into high-risk (NRG_score > median) and low-risk groups (NRG_score < median). The data was presented in a graphical format after principal component analysis (PCA) was conducted utilizing the package ”ggplot2”.

### Acquisition of Clinical Specimens, RNA Isolation, and Quantitative Real-Time Polymerase Chain Reaction PCR (RT-qPCR)

Six pairs of gliomas and non-tumor tissues adjacent to the tumors were collected from glioma patients at Zhongshan City People’s Hospital. The removed samples were immediately stored at -80°C until use. Informed consent was obtained from all patients with glioma participating in this research. The ethics committee of Zhongshan City People’s Hospital reviewed and approved the study.

Total RNA was extracted from glioma tissue using TRIzol reagent (Thermo Fisher Scientific, USA). RNA was reverse transcribed to Complementary DNA using HIScriptIIIRT SuperMix (Vazyme, Nanjing, China). The qRT-PCR analysis was performed using SYBR Green qPCR Master Mix (Vazyme, Nanjing, China) on ABI QuantStudioTM 5 (Agilent Technologies, USA). In all PCR experiments, data were quantified using the 2-ΔΔCt method and normalized by GAPDH. Primers for specific target genes (**Supplementary Table 4**) were synthesized by Genepharma Biotech (Shanghai, China).

To further assess the changes in protein levels of these genes, typical immunohistochemical results were obtained from the Human Protein Atlas (HPA) and analyzed for histochemistry score (H-SCORE). The staining intensity was divided into 4 grades: negative, grade 0; weakly positive, grade 1; positive, grade 2; strong positive, grade 3. Score H-SCORE according to the percentage of positive staining, H-SCORE = ∑ (Pixi) = (weakly positive Pi × 1) + (positive Pi × 2) + (strong positive Pi × 3) (where Pi represents the percentage of positive cells in the number of all cells in the section, and i represents the staining intensity; the total score range is 0 ~ 300).

### Survival, TMB, CSC, Mutation, Drug Sensitivity Analysis in High and Low-Risk Groups

We compared the difference of NRG_score in two groups. PCA analysis was applied to verify the differentiation of patients among the two groups. The receiver operating characteristic (ROC) and Kaplan-Meier survival analysis were applied for the prognostic prediction of patients. To assess the immune cells infiltrating the glioma, CIBERSORT was conducted. In addition, we evaluated the correlation of the 11 NRGs in the model with the infiltrating immune cells. The ESTIMATE package was applied to compare the TME between the two risk groups. We also evaluated the differences between TMB, gene mutations, PD-L1, PD-1, and CSC between the two risk groups. Using the “pRRophetic” package, semi-inhibitory concentrations (IC50) were calculated to assess the sensitivity of gliomas to common chemotherapy agents.

### The Development and Validation of a Nomogram

Using the ‘RMS’ package, we compiled the risk scores with the patient’s clinicopathological information and developed a predictive nomogram. In this nomogram, every variable corresponds to a score, and the total score is calculated by averaging all variables for one patient. The prediction power of the nomogram was assessed using time-dependent ROC curves for each dataset. To describe the accordance between the predicted and observed survival outcomes for 1-, 3-, and 5-years, calibration plots of the nomogram were analyzed. We also compared the accuracy of the WHO grade and the nomogram in predicting 1-, 3-, and 5-year survivals using time-dependent ROC curves.

### Statistical Analyses

The statistical analysis was conducted using R (4.1.0). A p-value or adjusted p-value of 0.05 is used as the level of statistical significance.

## Results

### The Landscape of Genetic and Transcriptional Variation of NRGs in Glioma

**Figure 2:**
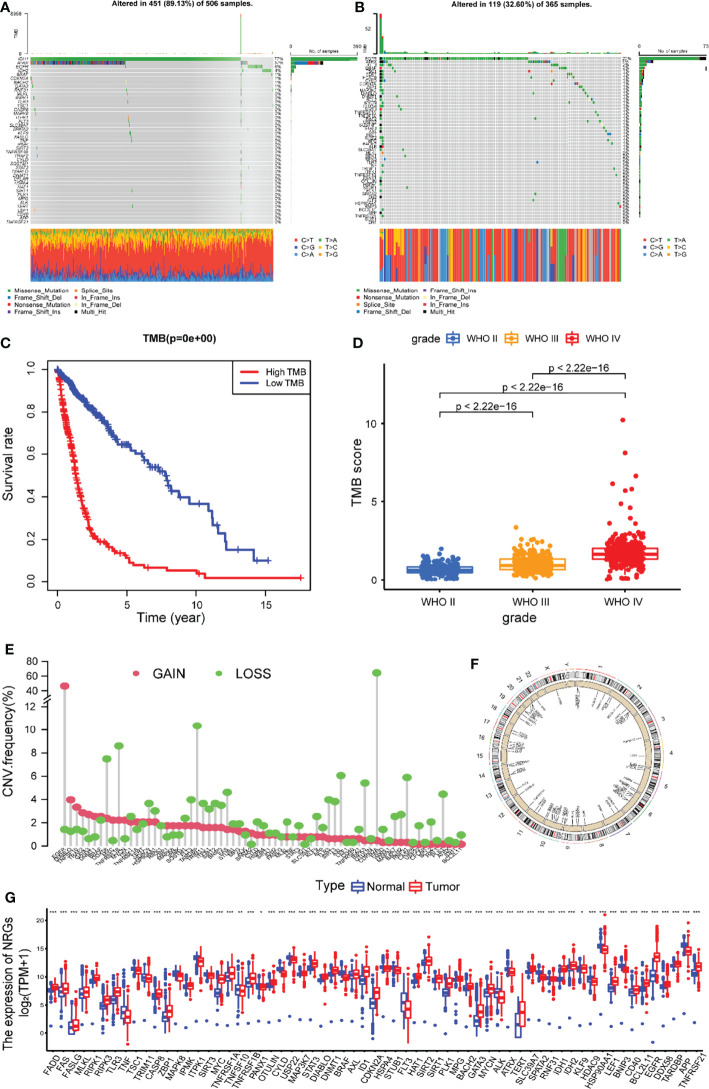
The landscape of genetic and transcriptional variation of NRG in glioma. Mutation frequencies of 67 NRGs in 506 patients with LGG **(A)** and 365 GBM patients without IDH1 mutation **(B)** from the TCGA dataset. **(C)** Kaplan-Meier survival analysis of the high TMB and low TMB groups. **(D)** Comparison of TMB score in gliomas of different grades. **(E)** The CNV variation frequencies of 67 NRGs in the patients with LGG or GBM from the TCGA dataset. **(F)** Locations of CNV alterations of 67 NRGs on 23 chromosomes. **(G)** The differential expression of 67 NRGs between gliomas and normal brain tissue. NRGs, necroptosis-related genes; LGG, low grade gliomas; GBM, glioblastoma; TCGA, The Cancer Genome Atlas; CNV, copy number variant; ***P<0.001, **P<0.01, *P<0.05.

The overview of the analysis of this research was presented in **Figure 1**. This study involved 67 NRGs in total. We first summarized the frequency of somatic mutations in these NRGs in glioma patients and found that LGG patients had a relatively high frequency of mutations (**Figure 2A**). As shown in **Figure 2A**, mutations occurred in 451 (89.13%) of 506 LGG samples, with missense mutations being the most common mutation classification. Among them, IDH1 had the highest mutation rate (77%), followed by ATRX (37%), EGFR (6%), and IDH2 (4%). As shown in **Figure 2B**, out of 365 samples of GBM patients without IDH1 mutation, 144 (32.60%) patients had mutations in NRGs, with EGFR having the highest mutation rate (20%), followed by ATRX (4%), FLT3 (2%) and BRAF (2%). Next, we explored the impact of mutations in these NRGs on the prognosis of glioma patients. As shown in **Figure 2C**, patients with a high tumor mutation burden (TMB) had significantly lower survival rates than those with lower TMB. Furthermore, the mutation frequency of NRGs gradually increased with increasing glioma grade (**Figure 2D**).

These NRGs were examined for the frequency of somatic copy number variation (CNV) in glioma from the TCGA cohort, and we found that CNV was frequent across all NRGs. The incidence of NRG deletion in glioma samples was mostly greater than the incidence of acquisition. Among them, CDKN2A, TARDBP, TNFRSF1B, BNIP3, SIRT3, and TLR3 had a higher incidence of loss, while EGFR, MYC, BRAF, TNFRSF1A, DIABLO, GATA3, and TNFSF10 had a higher incidence of CNV gain (**Figure 2E**). The location of these NRGs on chromosomes with altered CNVs is depicted in **Figure 2F**.

**Figure 3:**
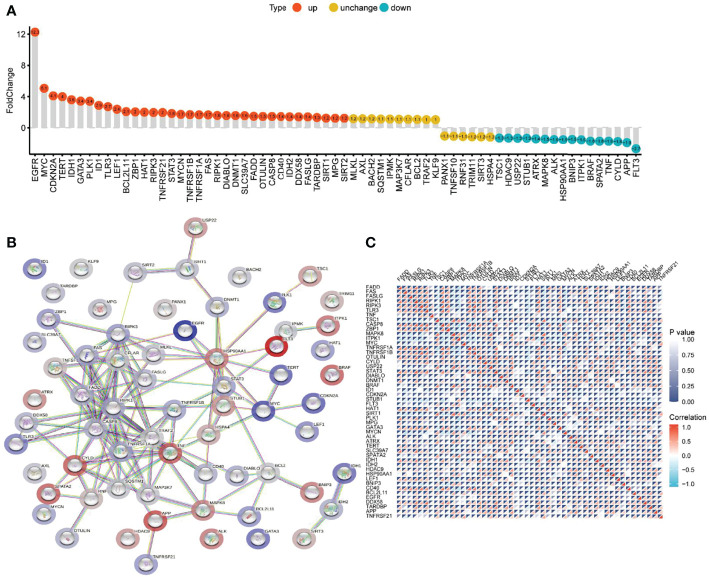
The differential expression, PPI network, and expression correlation of NRGs. **(A)** Differential expression of NRGs in normal brain tissue and glioma. **(B)**The PPI network of NRGs. **(C)** The correlation of expression of NRGs.

Comparing the differential expression of 67 NRGs in 1153 normal brain tissues (GTEx 1148, TCGA 5) with 698 glioma tissues (TCGA), we found 63 DEGs (all FDR <0.05, **Figure 2G**). As shown in **Figure 3A** and **Supplementary Table 5**, NRGs were categorized as highly expressed, lowly expressed, or not differentially expressed (|FoldChange| <1.2) in gliomas. Among them, 35 genes, including EGFR, MYC, CDKN2A, TERT, IDH1, GATA3, PLK1, ID1, and TLR3, were up-regulated; 16 genes, including TSC1, HDAC9, USP22, STUB1, ATRX, MAPK8, ALK, HSP90AA1, and BNIP3, were down-regulated; and the expression of 16 genes, including MLKL, AXL, BACH2, SQSTM1, IPMK, MAP3K7, CFLAR, and BCL2, was not significantly changed. To further explore the interactions between these NRGs, a PPI network was constructed. The result revealed that ZBP1, CASP8, CD40, CFLAR, CYLD, FAS, FADD, RIPK1, and RIPK3 were the hub genes (all combined score > 0.9) (**Figure 3B**). The relevant heatmap of the top 50 NRGs in TCGA was shown in **Figure 3C**. Our analysis revealed significant differences in genetic profiles and expression levels of NRGs between normal brain tissue and glioma, suggesting a potential role of NRGs in glioma.

### Identification of NRGclusters in Glioma

**Figure 4:**
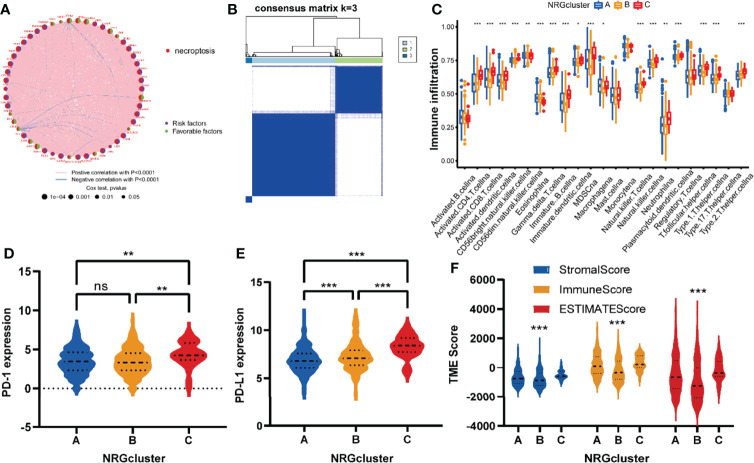
NRGclusters of gliomas divided by consensus clustering algorithm and clinicopathological characteristics of distinct subtypes. **(A)** Interactions of NRGs in glioma. The thickness of the line that connects a pair of NRGs indicates the strength of their association. Positive correlations are indicated in red and negative correlations in blue. **(B)** Heatmap of the consensus clustering matrix (k = 3) dividing glioma patients into the three NRGclusters. **(C)** The comparison of infiltration levels of immune cells in three NRGclusters. **(D, E)** Expression of PD-1 and PD-L1 in three NRG clusters. **(F)** Correlation between three NRGclusters and TME scores. TME, tumor microenvironment; ***P<0.001, **P<0.01, *P<0.05, ns P > 0.05.

To explore the important role played by NRGs in glioma patients, a comprehensive analysis was performed. Univariate regression analysis (**Supplementary Table 6**) showed that 50 of these NRGs had prognostic value (P<0.05). As shown in **Figure 4A** and **Supplementary Table 7**, NRGs interacted, co-expressed, and had a combined effect on the prognosis of glioma patients. Most of NRGs were positively correlated with each other and were risk factors.

Further investigating the role played by the NRGs in gliomas, we utilized a consensus clustering algorithm to examine subtypes of glioma patients according to the expression of NRGs (**Supplementary Figure 1A-F**). It appears that k = 3 is the best choice for classifying the dataset into NRGcluster A, B, and C (**Figure 4B**). In this scenario, the different subtypes are strongly correlated within groups and weakly correlated between groups.

Using ssGSEA to assess immune activity among glioma patients of different subtypes, we explored the role of necroptosis in glioma immunity. It was found that the infiltrating immune cells differed significantly between NRGclusters (**Figure 4C** and **Supplementary Table 8**). The infiltration level of immune cells was commonly high in NRGcluster C compared to the other two subtypes, especially activated CD8 T cells, activated dendritic cells, activated CD4 T cells, gamma delta T cells, MDSCs, immature B cells, natural killer cells, macrophages, and neutrophilia, etc.

Following this, we analyzed the immune checkpoints of these subtypes and found that both PD-1 and PD-L1 expression was higher in NRGcluster C than those in the other two subtypes (**Figures 4D, E**). TME scores of these NRGclusters were assessed utilizing the ESTIMATE, and similar to previous results, they were higher in NRGcluster C than those in the other two subtypes (**Figure 4F**).

### Identification of geneClusters Based on DEGs

**Figure 5:**
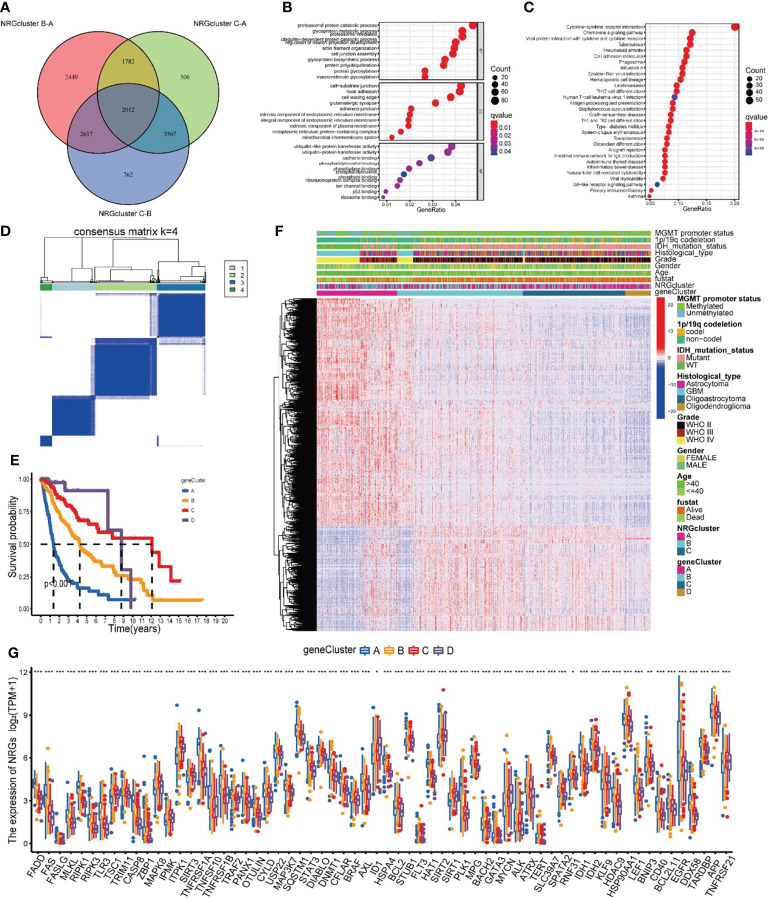
Identification of gene subtypes based on DEGs. **(A)** The Venn diagrams for the three NRGclusters. **(B, C)** Functional enrichment analysis of GO and KEGG for DEGs among three NRGclusters. **(D)** Heatmap of the consensus clustering matrix (k = 4) dividing glioma patients into four geneClusters. **(E)** Kaplan-Meier survival analysis of the four geneClusters. **(F)** The clinicopathologic features of the four geneClusters. **(G)** The differential expression of 67 NRGs among the geneClusters. DEGs differentially expressed genes; GO, Gene Ontology; KEGG, Kyoto Encyclopedia of Genes and Genomes; NRGs, necroptosis-related genes. ***P<0.001, **P<0.01, *P<0.05.

To further investigate the biological mechanisms underlying the differences in several NRGclusters, we applied the “limma” package to find DEGs of three subtypes and obtained 2012 DEGs by taking intersections (**Figure 5A**). The GO functional enrichment analysis of these DEGs revealed that they were enriched in biological processes related to immunity (**Figure 5B** and **Supplementary Table 9**). The KEGG analysis revealed that DEGs were enriched in pathways related to immune and inflammatory responses (**Figure 5C** and **Supplementary Table 9**). The previous results suggest that NRGs may be critical in tumor immunity. To further investigate the underlying regulatory mechanisms of NRGs, we initially conducted the univariate Cox regression analysis on 2012 DEGs and found that 1,866 of them were associated with prognosis (**Supplementary Table 10**). Then, based on these prognosis-related DEGs, a cluster analysis of samples was performed (**Supplementary Figure 2A–F**), and it was found that the samples of glioma patients in TCGA could be divided into geneClusters A-D 4 subtypes optimally (**Figure 5D**).

To verify the differences in prognosis across geneClusters, we performed survival analysis. According to the Kaplan-Meier curve, the prognosis of glioma patients in geneCluster A was worse than those in the other two subtypes (P < 0.001, **Figure 5E**). Subsequently, we compared the clinicopathological data and DEGs in patients of the 4 geneClusters. We found that poor prognosis in the geneCluster A group was associated with age > 40 years, WHO class IV, unmutated IDH, and unphosphorylated MGMT promoter (**Figure 5F**). In addition, the expression of NRGs was also generally significantly different between geneClusters(**Figure 5G**).

### Development and Validation of the Risk NRG_Score

**Figure 6:**
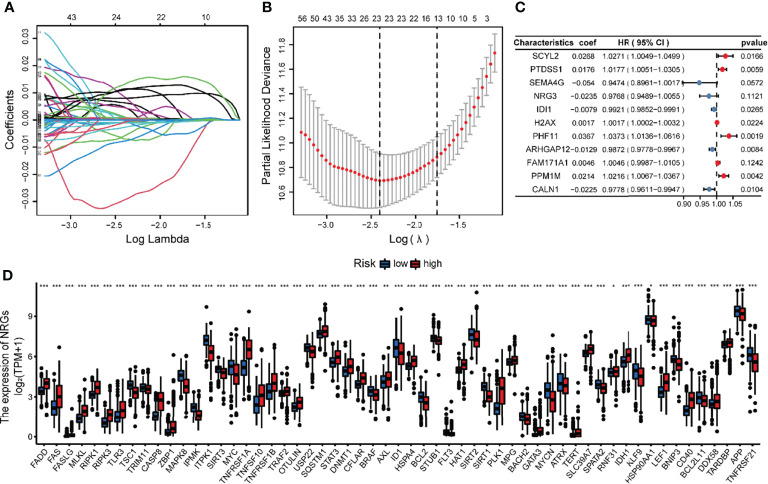
Identification of representative candidate prognostic genes and differential expression of NRGs between the high and low-risk groups. **(A)** The LASSO regression analysis of the candidate prognostic genes. **(B)** The partial likelihood of deviance of the prognostic genes. **(C)** Forest plot of the multivariate Cox regression analysis for candidate genes. **(D)** The differential expression of NRGs between the high and low-risk groups. ***P<0.001, **P<0.01, *P<0.05.

NRG_score was constructed based on the prognosis-related DEGs of different subtypes. We divided the TCGA glioma patients into training (n = 444) and validation sets (n = 218) in the ratio of 2:1, utilizing the package “caret”. We then applied Lasso regression analysis to screen out the optimum 23 prognostic genes among the DEGs (**Figures 6A, B**). The Subsequent multivariate Cox regression analysis was conducted on them, and we finally got 11 genes, including 5 low-risk genes (SEMA4G, NRG3, IDI1, ARHGAP12, CALN1) and 6 high-risk genes (SCYL2, PTDSS1, H2AX, PHF11, FAM171A1, PPM1M) (**Figure 6C**). According to the multivariate cox regression results, the formula of NRG_score was constructed as follows: risk score = (0.0268*expression of SCYL2) + (0.0176*expression of PTDSS1) + (-0.0540*expression of SEMA4G) + (-0.0235*expression of NRG3) +(-0.0079*expression of IDI1) +(0.0017*expression of H2AX) + (0.0367*expression of PHF11) + (-0.0129*expression of ARHGAP12) + (0.0046*expression of FAM171A1) + (0.0214*expression of PPM1M) + (-0.0225*expression of CALN1).

**Figure 7:**
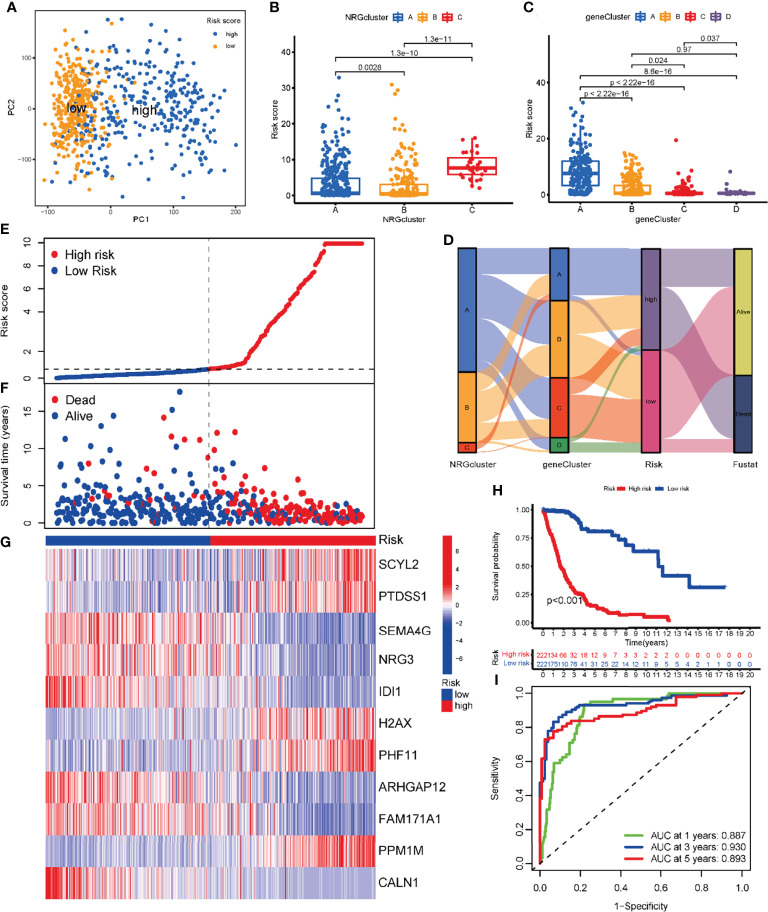
Development of the risk NRG_score in the training dataset. **(A)** PCA analysis based on the NRG_score. The blue and yellow dots represent the high- and low-risk groups, respectively. **(B)** Comparison of NRG_score in different NRGclusters. **(C)** Comparison of NRG_score in different geneClusters. **(D)** Alluvial diagram of the distribution of subtypes with different NRG_score and survival outcomes. **(E)** The ranked dot of the distribution of the NRG_score. **(F)** Scatter plots of the distribution of the survival status in patients from high- and low-risk groups. **(G)** The expression of genes in the NRG_score model in patients from two risk groups. **(H)** Kaplan-Meier survival analysis of the two risk groups. **(I)** ROC curves of the NRG_score model for predicting the sensitivity and specificity of 1-, 3-, 5-year survival in patients with glioma.

The glioma patients of TCGA were assigned to high-risk (NRG_score > median, n = 222) and low-risk (NRG_score < median, n = 222) groups. The expression of most NRGs is significantly different between the high and low risk groups (**Figure 6D**). The PCA analysis (**Figure 7A**) showed that NRG_score was able to discriminate patients well.

We explored the distribution of NRG_score among different subtypes. The NRG_scores of patients in three NRGclusters are statistically significantly different (P<0.01) (**Figure 7B**). Similarly, the NRG_score also differed significantly among several geneClusters (**Figure 7C**), with geneCluster A patients having the highest risk score, geneCluster C having the lowest risk score, and geneClusters B and D in the middle. Next, we examined the distribution of patients within the NRGcluster groups, the geneCluster groups, and the NRG_score risk groups (**Figure 7D**).

Based on the previous results, we found that the higher the NRG_score, the lower the survival rate of glioma patients. Further validating the result, we ranked all patients according to the NRG_score. According to the NRG risk distribution plot, the number of patients who died increased with increasing risk scores, and most of these deaths were attributed to patients with high-risk scores (**Figures 7E, F**). We present the expression level of the genes involved in the risk model (**Figure 7G**). In the high-risk group, glioma patients had a lower survival rate than that in the low-risk group (P < 0.001; **Figure 7H**). In addition, the NRG risk score had high prognostic predictive performance, with AUC values of 0.887, 0.930, and 0.893 for 1-, 3- and 5-year survival, respectively (**Figure 7I**).

To further validate the excellent prognostic predictive performance of NRG_score, we utilized the same formula to calculate the risk scores of patients in the internal test dataset and two external validation sets of CGGA and Rembrandt, and we divided the patients into two risk groups. We found that the number of patients who died increased significantly with increasing NRG_score in the internal test group (**Supplementary Figure 3A**), the two external validation sets of the CGGA (**Supplementary Figure 3B**), and Rembrandt (**Supplementary Figure 3C**). According to the survival analysis of the three validation sets (**Supplementary Figures 3D–F**), the survival rate of glioma patients was higher in the low-risk group than that in the high-risk group (P<0.001). The analysis of the predictive prognostic performance of NRG risk score (**Supplementary Figures 3G–I**) found that the AUC values of 1-, 3-, and 5-year survival remained high for the three validation sets, and the apparent NRG risk scores had excellent predicting prognostic power.

### Validation of Expression Levels of 11 Genes in the Prognostic Model

**Figure 8:**
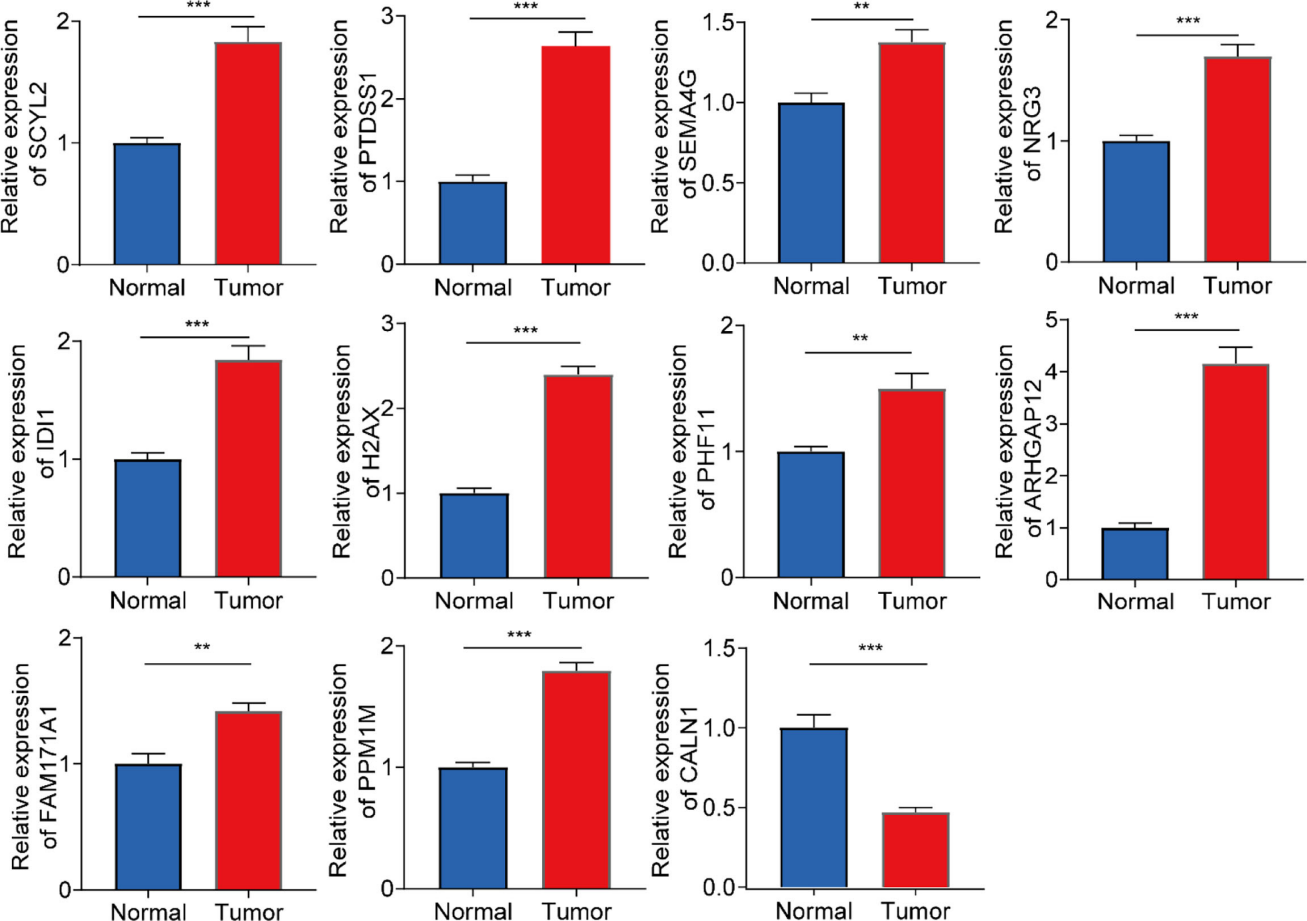
RT-qPCR analysis on the expression levels of 11 genes in the prognostic model in 6 pairs of gliomas and their adjacent normal tissues. ***P<0.001, **P<0.01.

To further evaluate the expression levels of genes in the model in clinical samples, we subjected 6 pairs of gliomas and their adjacent normal tissues to RT-qPCR analysis. As shown in **Figure 8**, 11 genes were significantly differentially expressed in glioma and adjacent normal brain tissues. In glioma tissues, most genes were upregulated in expression levels, while CALN1 expression was downregulated.

**Figure 9:**
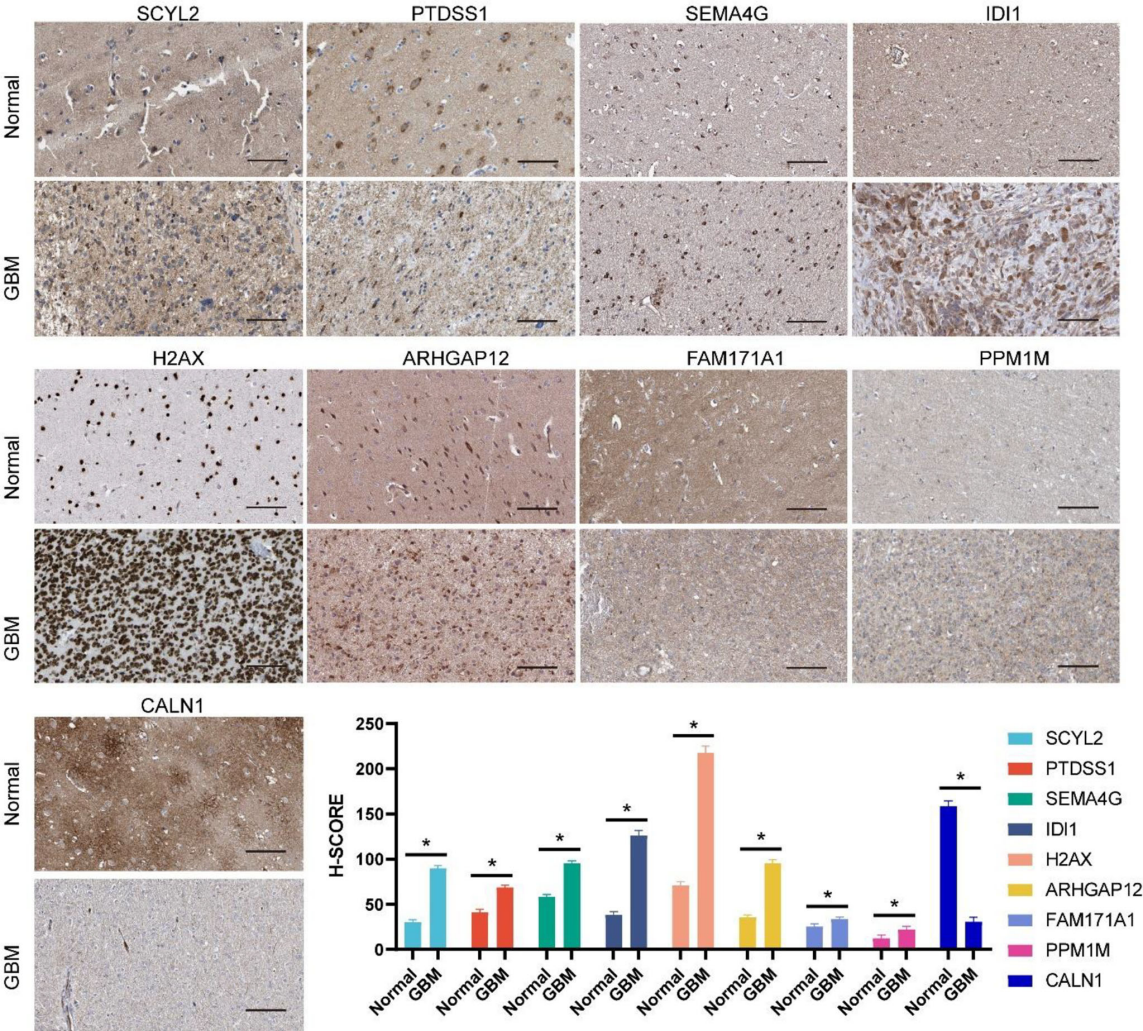
Protein expression levels of genes incorporated in the prognostic model between normal brain and glioma tissue as demonstrated by immunohistochemical analysis and the H-SCORE analysis. Bar = 100µm. *p < 0.05.

To further assess the changes in protein levels of these genes, typical immunohistochemical results obtained from the Human Protein Atlas (HPA) were presented. Among them, NRG3 and PHF11 protein expression data were not available in HPA. As shown in **Figure 9**, the differential trends in protein expression levels of the other nine genes were approximately the same as those in the mRNA expression levels. The above results verified the expression changes of 11 genes in the model at mRNA and protein levels and laterally confirmed the reliability of the genes included in the model.

### Comparison of TME and Checkpoints Between the Two Risk Groups

**Figure 10:**
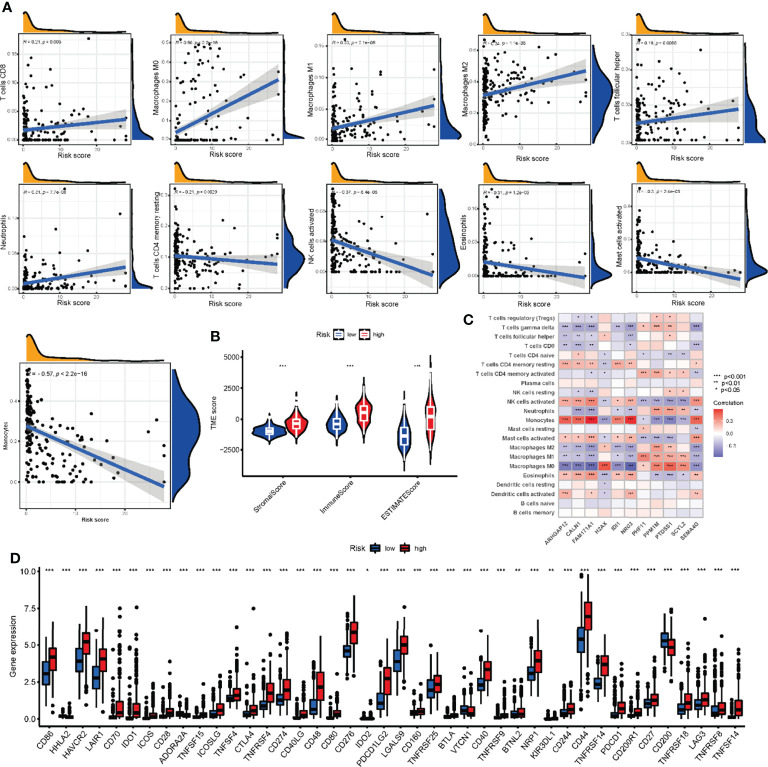
Comparison of TME and checkpoints between the high- and low-risk groups. **(A)** Correlations between infiltrating immune cells and NRG_score. **(B)** Correlations between TME scores and NRG_score. **(C)** Correlations between the abundance of infiltrating immune cells and expression of NRGs in risk score model. **(D)** Expression of immune checkpoints between the two risk groups. TME, tumor microenvironment; NRGs, necroptosis-related genes. ***P<0.001, ** P<0.01, *P<0.05.

To assess the correlation between NRG scores and TME, we utilized the CIBERSORT algorithm to assess the level of immune cells infiltration. The NRG_score was positively correlated with CD8 + T cells, M1 macrophages, M0 macrophages, M2 macrophages, activated NK cells, follicular helper T cells, and neutrophils, and negatively correlated with CD4 memory resting T cells, Eosinophils, activated mast cells, and monocytes (**Figure 10A**). We also found that the high-risk group had significantly higher TME Scores, reflecting a higher level of immune cells infiltration (**Figure 10B**).

In addition, we evaluated the correlation between the expression of 11 genes in the risk model and the abundance of immune cells. There was a significant correlation between the expression of 11 genes and immune cells (**Figure 10C**). In addition, 33 immune checkpoints including PD-L1, PD-1, and CTLA-4 were differentially expressed in the two groups (**Figure 10D**).

### Correlation Between NRG_Score and Mutation, CSC, and Drug Sensitivity

**Figure 11:**
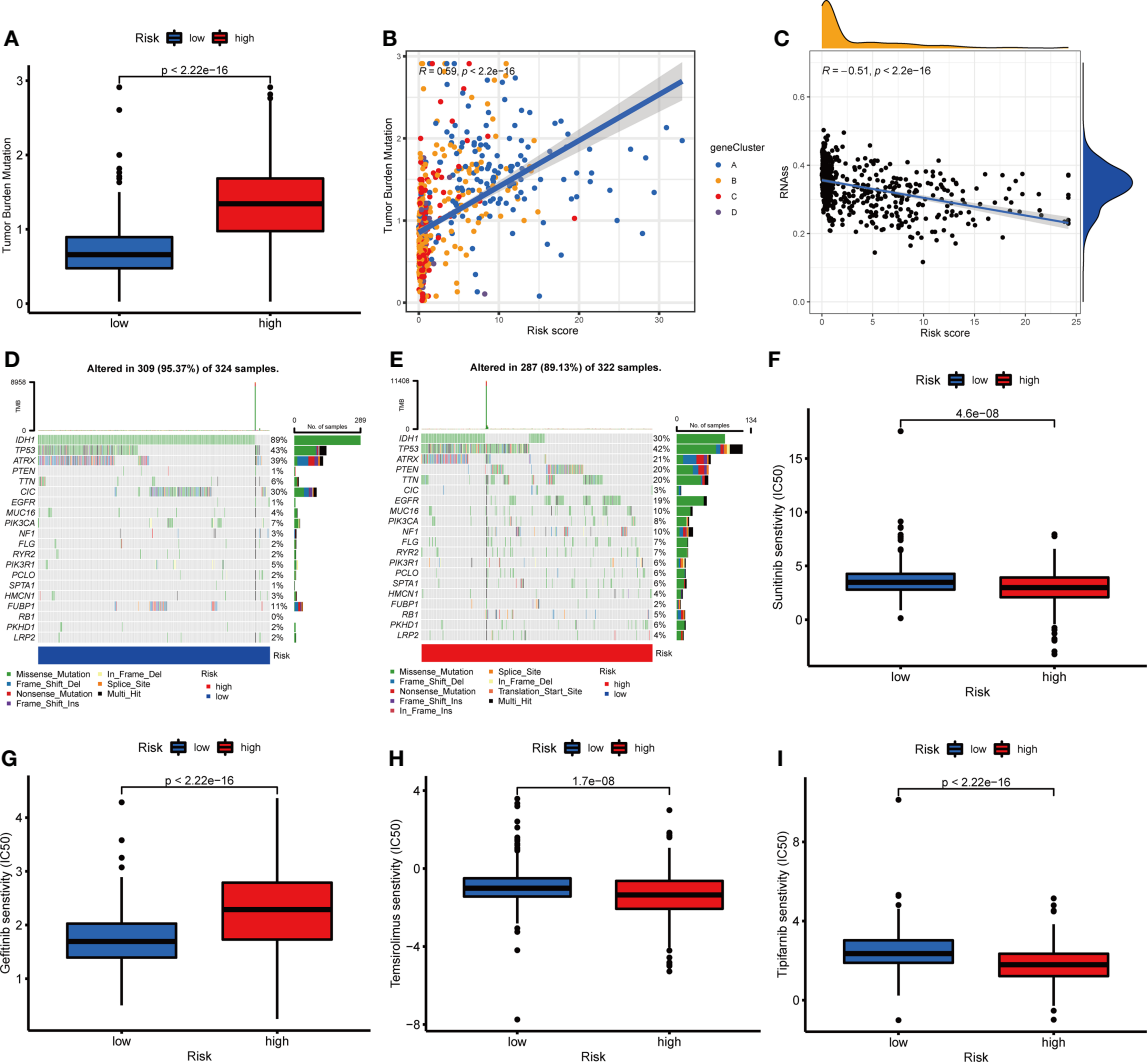
Correlation between NRG_score and mutation, CSC index, and drug sensitivity. **(A)** Comparison of TMB in two risk groups. **(B)** Spearman correlation analysis between TMB and the NRG_score. **(C)** Spearman correlation analysis between CSC index and the NRG_score. **(D, E)** The waterfall plot of somatic mutation features in the low- **(D)** and high-risk **(E)** groups. **(F–I)** Comparison of chemotherapy sensitivity in glioma between two risk groups.

To further evaluate whether glioma patients could benefit from immunotherapy, we conducted a tumor mutation analysis. According to the results, a significantly higher TMB was observed in the high-risk group than that in the low-risk group (**Figure 11A**), indicating that immunotherapy was more likely to be effective in patients in the high-risk group. According to Spearman correlation analysis, TMB correlated positively with NRG_score (R=0.59, p<0.001, **Figure 11B**). Following this, we examined the correlation between CSC and NRG_score, and found it to be negative (R=-0.51, p<0.001, **Figure 11C**), suggesting low tumor stem cell characteristics and high differentiation in patients with high NRG_score. Furthermore, we examined the distribution of somatic mutations between two risk groups. We found that IDH1, CIC, FUBP1, and ATRX mutation frequencies were significantly higher in patients of the low-risk group than those in patients of the high-risk group, while PTEN, TTN, EGFR, and MUC16 genes were mutated more frequently in patients of the high-risk group (**Figures 11D, E**).

We then evaluated the predictive value of NRG_score on patients’ chemotherapeutic drug sensitivity. We found that the IC50s for angiogenesis inhibitors such as sunitinib, and sorafenib were lower in patients of the high-risk group than those in patients of the low-risk group (**Figure 11F** and **Supplementary Figure 4A**). For the commonly used EGFR tyrosine kinase inhibitors, the sensitivity differed between the risk groups. Compared to the low-risk group, patients in the high-risk group had lower IC50 values for Erlotinib (**Supplementary Figure 4B**) and higher IC50 values for Gefitinib and Nilotinib(**Figure 11G** and **Supplementary Figure 4C**). In addition, the IC50 values of Temsirolimus (**Figure 11H**), Tipifarnib (**Figure 11I**), Bicalutamide (**Supplementary Figure 4D**), Gemcitabine (**Supplementary Figure 4E**), Parthenolide (**Supplementary Figure 4F**), Salubrinal (**Supplementary Figure 4G**), and vinblastine (**Supplementary Figure 4I**) were lower in the patients of the high-risk group than those of the low-risk group, while IC50 value of shikonin (**Supplementary Figure 4H**) was higher in patients of the high-risk group than that in the low-risk group. It appears that the NRG_score may be used to predict patients’ drug sensitivity, even in the sense of guiding the selection of different classes of drugs in the same category.

### Development and Validation of a Nomogram to Predict Survival

**Figure 12:**
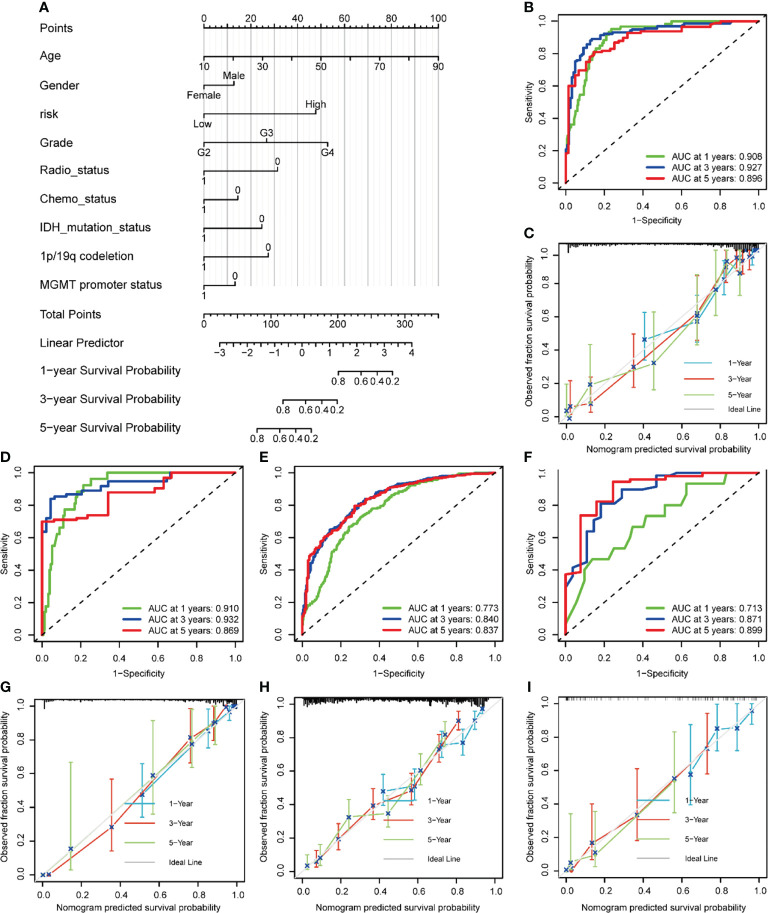
Construction and validation of a nomogram to predict survival of patients with glioma. **(A)** Nomogram for predicting the 1-, 3-, 5-year survival of glioma patients in the training set. **(B)** ROC curves of the nomogram for predicting the sensitivity and specificity of 1-, 3-, 5-year survival in glioma patients in the training dataset. **(C)** Calibration curves of the nomogram for predicting 1-, 3-, and 5-year survival in glioma patients in the training dataset. **(D-F)** ROC curves of the nomogram for predicting the sensitivity and specificity of 1-, 3-, 5-year survival in glioma patients in the TCGA testing dataset(D), CGGA **(E)**, and Rembrandt **(F)**. **(G-I)** Calibration curves of the nomogram for predicting 1-, 3-, 5-year survival in glioma patients in the TCGA testing dataset **(G)**, CGGA **(H)**, Rembrandt **(I)**.

To make the NRG_score easier to apply clinically, we combined it with the clinicopathology of glioma patients to develop a nomogram for predicting patients’ prognosis (**Figure 12A** ands **Supplementary Table 11**). The AUC values of the nomogram for predicting the 1-year, 3-year, and 5-year survival rates of the training set were 0.908, 0.927, and 0.896, respectively (**Figure 12B**), and the calibration curves also suggested high accordance between the predicted probabilities and the observed probabilities, indicating the high reliability of the nomogram (**Figure 12C**). Similarly, we also demonstrated high accuracy of nomogram in predicting 1-, 3-, and 5-year survival with high reliability in an internal validation set (**Figure 12D**), and two external validation sets (CGGA (**Figure 12E**), and Rembrandt (**Figure 12F–I**).

Subsequently, we compared the predictive accuracy of our nomogram with that of the WHO-grade in the training and validation datasets. We found that the AUC values of 1,3,5-year survival (**Supplementary Figures 5A, E, I**) were higher than those predicted by WHO grade in the training dataset. The same results were obtained in the internal validation dataset (**Supplementary Figures 5B, F, J**), the CGGA external validation set (**Supplementary Figures 5C, G, K**), and the Rembrandt external validation set (**Supplementary Figures 5D, H, L**), indicating that the nomogram has better predictive performance than that of the WHO grade.

## Discussion

Targeting necroptosis has also emerged as a promising therapeutic approach to bypass apoptosis resistance and support anti-tumor immunity. However, the role played by necroptosis-related genes in the development and prognosis of glioma has not been effectively elucidated by current studies. Ferroptosis-related genes (33), pyroptosis-related genes (34), and autophagy-related genes (35) have been reported in the literature for predicting survival in patients with glioma. However, the effect of necroptosis-related genes (NRGs) on predicting the survival of patients with gliomas and on the tumor microenvironment in gliomas has not been reported. Therefore, our study still has some theoretical and clinical significance.

In this study, we first analyzed the overall variation of 67 NRGs at the genetic and transcriptional levels in glioma patients. Based on candidate NRGs, we divided patients into 3 NRGclusters and 4 geneClusters. To further investigate the potential role of NRGs in regulating immunity in patients with glioma, we found that the level of immune cells infiltration and TME scores were generally higher in geneCluster C than those in the other subtypes. Immune checkpoint comparison also revealed that PD-L1 and PD-1 were higher in geneCluster C than those in the other subtypes.

To further investigate the biological behavior behind the differences in several NRGclusters, we screened the differential genes and performed GO, KEGG functional enrichment analysis, and found that DEGs were enriched in immune and inflammation-related pathways, indicating that NRGs are indispensable for these processes.

As well, we conducted the univariate Cox regression, Lasso regression, and multivariate Cox regression analysis on the DEGs to screen out prognosis-related genes, and four geneClusters were identified based on the consensus clustering analysis. A survival analysis revealed significant differences in prognosis among the four subtypes. The patients were randomly assigned to training and validation groups. And by lasso regression and multivariate cox regression analysis, we finally screened for 11 genes to construct a risk score model. It was found that the model had excellent predictive performance when it came to predicting the prognosis of patients in these datasets.

Moreover, the risk score correlated and predicted well for treatments such as TMB, immune checkpoint, immune cell infiltration, and CSC. Furthermore, the risk score can be used as a guide in predicting the sensitivity to chemotherapy drugs. To facilitate more clinical application, we combined the NRG_score with clinical information and developed a nomogram to predict the prognosis of patients with glioma in datasets. ROC curves and calibration plots demonstrated that the nomogram was highly predictive and reliable. Moreover, the predictive powers of the nomogram were more accurate than those of the WHO grade. As such, the nomogram may be applied to predict the prognosis of patients suffering from gliomas. The study makes it clear that NRGs are indispensable to glioma progression, immunomodulation, and treatment, and that they can provide a guide for sensitive drug screening during chemotherapy, which could provide new ideas for individually targeted therapies.

We screened for DEGs between a normal brain and glioma tissue from 67 NRGs and constructed PPI networks to explore hub genes. It was found that ZBP1, CASP8, CD40, CFLAR, CYLD, FAS, FADD, RIPK1, and RIPK3 were the hub genes. Yang et al. (36) reported that the DNA-dependent activator of IFN regulatory factors (ZBP1) plays an important role in IFN signaling during anti-necrotic apoptosis. ZBP1 is involved in regulating RIPK1/RIPK3-FADD-caspase-8 cell death complex assembly, along with their important regulatory role in necroptosis (37, 38). Osborn et al. (39) reported that the Fas-associated death domain (FADD), a negative regulator of necroptosis, plays a vital role in T cell receptor-mediated necroptosis.

By inhibiting necroptosis, caspase-8 also contributes to survival (40–42). And it has been reported (43) that inhibition of caspase-8 leads to CYLD-dependent necroptosis. He et al. (44)reported that the anti-apoptotic protein CFLAR (CASP8 and FADD-like apoptosis regulator) plays a key role in necroptosis in T lymphocytes. A large body of literature reports that RIPK acts as an important sensor, receiving stimuli from both inside and outside the cell, and is involved in several biological processes such as immune response, inflammation, and cell death; RIPK1 and RIPK3 are essential for necroptosis (7, 13, 14, 45).

Correlation analysis showed that the expression of numerous NRGs was positively correlated and most of them were risk factors, suggesting a common role in regulating necroptosis. In contrast to previous studies focusing on a single gene, it is equally important to investigate the role played by the gene set as a whole. Based on the consensus clustering analysis, glioma patients were divided into three subtypes (43, 44).

We performed two layers of clustering, the first layer is NRGcluster, and the second layer is geneCluster. The main purpose of NRGcluster is to find the number of clusters with the strongest intra-group correlation and the weakest inter-group correlation. The second layer of clustering builds on the first layer of clustering to find the differentially expressed genes among different NRGclusters for a deeper geneCluster. For a deeper understanding of the differences among these NRGclusters, we performed enrichment analyses of DEGs within each subtype. GO and KEGG results also revealed substantial enrichment in immune and inflammation-related biological pathways, which suggested that NRG may be indispensable for the regulation of tumor immunity.

The consensus clustering analysis was conducted on prognosis-related DEGs, and several geneClusters were finally derived. The prognosis and expression of NRGs differed significantly among the geneClusters. To facilitate generalized application, we developed an NRG_score risk prediction model and confirmed the high predictive efficacy and reliability of the model in all datasets. NRG_score was used to categorize patients into high- and low-risk groups. By comparing the expression of NRGs in two risk groups, we found that the expression of NRGs such as ZBP1, RIPK1, RIPK3, FADD, FAS, and MLKL was widely lower in the low-risk group than those in the high-risk group, suggesting that the risk score can well reflect the incidence of necroptosis.

To investigate the mechanisms of immune modulation by NRGs, we performed a comparative analysis of the infiltration of immune cells, immune checkpoints, TMB, and CSC in the two risk groups. High-grade gliomas have a poor prognosis, and although some patients respond better to radiotherapy treatment, tumor heterogeneity is high. This highlights the critical role of TME in glioma development and progression. Targeting the heterogeneity of glioma TME provides new ideas for the treatment of glioma (46). There is a combination of immune cells, stromal cells, fibroblasts, endothelial cells, cellular stroma, and blood vessels surrounding the TME, which are responsible for the heterogeneity of the tumor as well as for its development and prognosis (47). It was found that our NRG_score was significantly related to the infiltration of immune cells. A positive correlation was found between NRG_score and CD8 + T cells, M1 macrophages, follicular helper T cells, M2 macrophages, and neutrophils, M0 macrophages, and a negative correlation was found with activated NK cells, Eosinophils, activated mast cells, CD4 memory resting T cells, and Monocytes.

Monocytes perform a crucial role in immune surveillance and immunological response regulation (48, 49). We found that necrotizing apoptosis was negatively correlated with monocyte expression. This may be because monocytes migrate to the tumor region during pathological situations, such as necroptosis in glioma, and then differentiate into immunological cells, such as macrophages. It has been reported (46) that monocyte-derived macrophages are essential in the regulation of the microenvironment of brain tumors, which supports our findings. Macrophages (Mφs) generally regulate immune responses and ultimately maintain immune homeostasis through biological processes such as pathogen phagocytosis and antigen presentation (50). Mφs can be simplified as inflammatory M1 or immunosuppressive M2 Mφs (51), and the M1, and M2 of Mφ are convertible between them and exist continuously and uniformly (52). Our study found that the infiltration of several subtypes of macrophages was elevated in the high-risk group of gliomas. Moreover, the pro-inflammatory and immunosuppressive functions of each macrophage were again uniformly present, which laterally reflects part of the source of glioma tumor heterogeneity. Follicular helper T cells are a class of CD4+ T cell subpopulations (53), whose main function is to assist B cells in humoral immunity and enhance the immune response (54). High CD8+ T cells infiltration is also a sign of high immune response. We found a higher incidence of necroptosis and a higher immune response in the high-risk group than those in the low-risk group. There is also evidence (8) suggesting that exposure to necrotizing apoptotic cells within the TME is associated with an increased number of tumor-specific CD8+ T cells within the tumor tissue. This is in accordance with our results. NK cells, as innate immune cells, can target tumor cells and perform a crucial role in tumor detection, elimination of malignant cells, and limiting tumor metastasis (55, 56). The negative association of activated NK cells with risk scores found in our study is consistent with previous studies. Consistent with the results of increased immune cell infiltration, the TME scores were higher in the high-risk group than those in the low-risk group, and the expression of genes in the model was most significantly correlated with immune cell expression. It is suggested that the immune microenvironment can be scored and predicted by the correlation between NRG_score and immune cell infiltration in the risk model.

With the development of immunology and molecular biology for tumors, immune checkpoint-based immunotherapy provides a new idea for tumor treatment. The PD-L1 and PD-1 perform a vital role in tumorigenesis and progression and could be a target for tumor immunotherapy. It has been reported that PD-1 and PD-L1 exert a vital role in the progression and immunotherapy of glioma (57). According to Baral et al., The expression of PD-L1 correlates with the WHO grade in gliomas. The expression of PD-L1 is common in GBM but is mostly restricted to a small subpopulation of infiltrating T cells, forming a “molecular barrier” that contributes to tumor immune escape and promotes tumor malignancy (58). A high PD-L1 expression is related to prognosis, and therefore screening out the Tipifarnibpatients with high PD-L1 expression is important for predicting good immunotherapy outcomes. Consistent with previous findings, we also found that patients with glioma had higher PD-1, PD-L1, and worse prognosis in the high-risk group than those in the low-risk group. Our risk score model can also assist in predicting PD-L1 expression, which is valuable as a guide to screening out patients with good responses to PD-1 immunotherapy.

TMB could be utilized to predict the survival of patients after treatment with immune checkpoint inhibitors (ICIs) in a variety of cancers. Most patients with tumors with a higher TMB, except for gliomas, will likely benefit from ICI therapy and have a better prognosis. Generally, patients with higher TBM gliomas often have a worse prognosis (59). These patients’ poor prognosis is most likely due to their own high WHO grade. A higher TMB in these patients may be the result of their previous exposure to temozolomide, which may lead to the development of less immunogenic subclonal mutations (60). In addition, we found that patients in the high-risk group had higher TBM values and that the TMB and the NRG_score were positively related. Additionally, the NRG_score and CSC showed a negative correlation. These studies suggest that patients with high-risk scores have malignantly differentiated tumor cells with inherently high heterogeneity, which may also result in different responses to antitumor immunotherapy and low dependence on TMB. Therefore, the response of patients to PD-1 and TMB may not always be consistent.

We further investigated the tumor mutations in two risk groups. We found a lower frequency of IDH1 and CIC mutations and a higher frequency of PTEN, EGFR, and TTN mutations in patients from the high-risk group than those from the low-risk group. Zhang et al. (61) reported that IDH and CIC mutations occurred primarily in low-grade gliomas and that they were associated with a significantly higher survival rate. In contrast, EGFR, PTEN, and TTN were related to worse survival. This is in accordance with our findings.

From the above results, we speculated that patients screened from the high-risk group may be easier to benefit from multiple drugs such as immunotherapy than patients in the low-risk group. Thus, we assessed the patients’ sensitivity to currently used chemotherapy and immunotherapy drugs. For angiogenesis inhibitors, the patients in the high-risk group were more likely to benefit from sunitinib and sorafenib than from other similar drugs. And among PI3K-Akt-mTOR pathway inhibitors, Temsirolimus was preferred over Everolimus for patients with high-risk glioma. Tipifarnib and Lonafarnib are a class of Farnesyl transferase inhibitors that induce death in radiotherapy-insensitive gliomas (62–64). We also found that patients in the high-risk group were more sensitive to Tipifamib than Lonafarnib. Interestingly, for the commonly used epidermal growth factor receptor tyrosine kinase inhibitors, patients in the high-risk group were more sensitive to Erlotinib, while the low-risk group should prefer the highly sensitive Gefitinib and Nilotinib. In addition, Bicalutamide, Gemcitabine, Parthenolide, Salubrinal, vinblastine, and Shikonin have also been reported for the treatment of glioma (65–69). Patients in the high-risk group may benefit more from Bicalutamide, Gemcitabine, Parthenolide, Salubrinal, and vinblastine; While in the low-risk group, the patients were more sensitive to shikonin. The results revealed that NRG_score can be used to predict the drug sensitivity of patients and even provide guidance in the selection of different drugs in the same class.

To facilitate clinical application, we combined NRG_score with clinical data to develop a nomogram for predicting the prognosis of patients. In the training set, the internal validation set, and both external validation sets of CGGA and Rembrandt, the model proved to have excellent predictive efficacy and high reliability.

Our study has several limitations. First, there are not many studies on necroptosis, especially its study in glioma is even less. The selected NRGs may only be the tip of the iceberg, and more basic studies are needed to confirm more necroptosis molecules. Secondly, we chose all public data from public databases; it would be more convincing to use prospective own data.

## Conclusions

We integrated the broad impact of NRGs on tumor immunity, TME, and prognosis at genetic and transcriptional levels. We constructed risk scores and a nomogram for the survival prediction of patients with glioma. The findings of this research provide new insights into personalized targeted therapies for gliomas.

## Data Availability Statement

The datasets presented in this study can be found in online repositories. The names of the repository/repositories and accession number(s) can be found in the article/**Supplementary Material**.

## Ethics Statement

The identification of necroptosis-related subtypes, the construction of a prognostic model, and the characterization of the tumor microenvironment in gliomas were reviewed and approved by Zhongshan City People’s Hospital. The patients/participants provided their written informed consent to participate in this study.

## Author Contributions

CL, JG, and YG conceived the project. YB, JS, and SG contributed to data analysis and interpretation, and manuscript writing and revision. ZL, ZW, and CC contributed to data acquisition and conducted the experiments. All authors read and approved the submitted manuscript.

## Funding

Funding for this research was provided by the Science and Technology Program of Guangzhou, China under Grant to YG (Grant No. 201604020080); by the Guangdong Basic and Applied Basic Research Foundation under Grant to YG (Grant No. 2018B0303110014 and No. 2020B090924004), and by the Natural Science Foundation of Guangdong under Grant to Jin Gong (Grant NO. 2021A1515010464).

## Conflict of Interest

The authors declare that the research was conducted in the absence of any commercial or financial relationships that could be construed as a potential conflict of interest.

## Publisher’s Note

All claims expressed in this article are solely those of the authors and do not necessarily represent those of their affiliated organizations, or those of the publisher, the editors and the reviewers. Any product that may be evaluated in this article, or claim that may be made by its manufacturer, is not guaranteed or endorsed by the publisher.
