# Supplementary material for: The Identification of Necroptosis-Related Subtypes, the Construction of a Prognostic Model, and the Characterization of the Tumor Microenvironment in Gliomas
Source: Front Oncol. 2022 Jun 2;12:899443. doi: 10.3389/fonc.2022.899443 (PMC9231435; doi:10.3389/fonc.2022.899443)
Supplement: Supplementary file 1 [file Image_1.pdf]

Supplementary Material

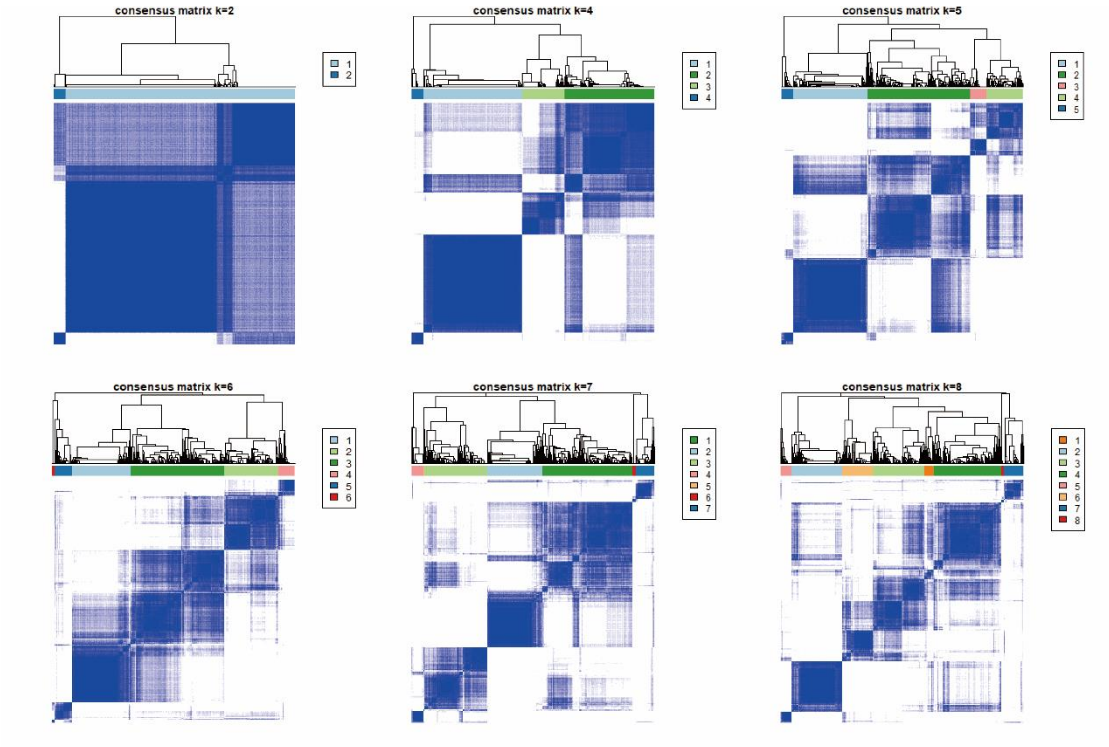

Supplementary Figure 1. (A-F) Heatmap of the consensus clustering matrix (k = 2, k=4-8) dividing glioma patients into different NRGclusters.

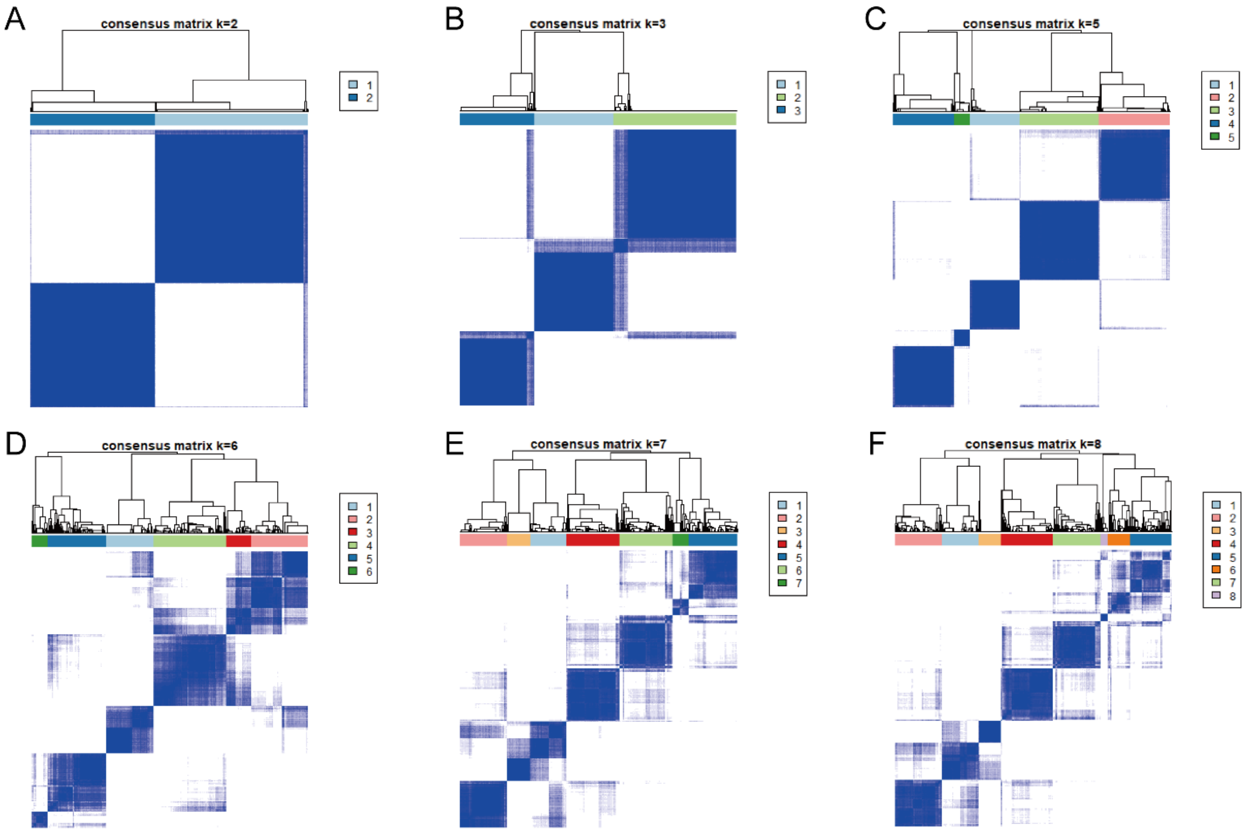

Supplementary Figure 2. (A-F) Heatmap of the consensus clustering matrix (k = 2-3, k=5-8) dividing glioma patients into different geneClusters.

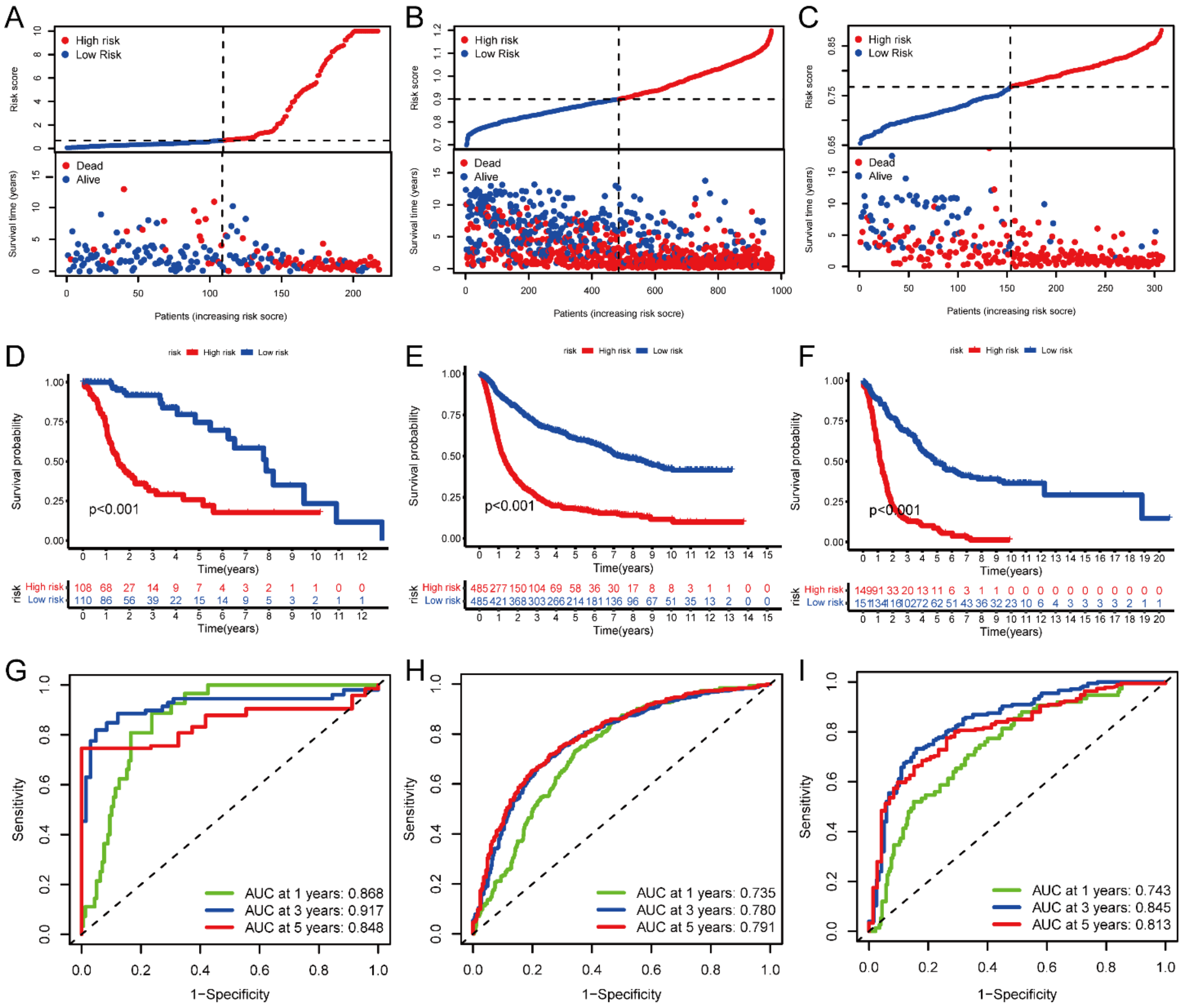

Supplementary Figure 3. Validation of NRG_score in the testing datasets. (A-C) The Ranked dot and scatter plots of the distribution of the NRG_score and survival status in glioma patients from the testing dataset of TCGA (A), CGGA (B), and Rembrandt (C). (D-F) Kaplan-Meier survival analysis of the two risk groups in glioma patients from the testing dataset of TCGA (D), CGGA (E), and Rembrandt (F). (G-I) ROC curves of the NRG_score model for predicting the sensitivity and specificity of 1-, 3-, 5-year survival in glioma patients from the testing dataset of TCGA (G), CGGA (H), and Rembrandt (I).

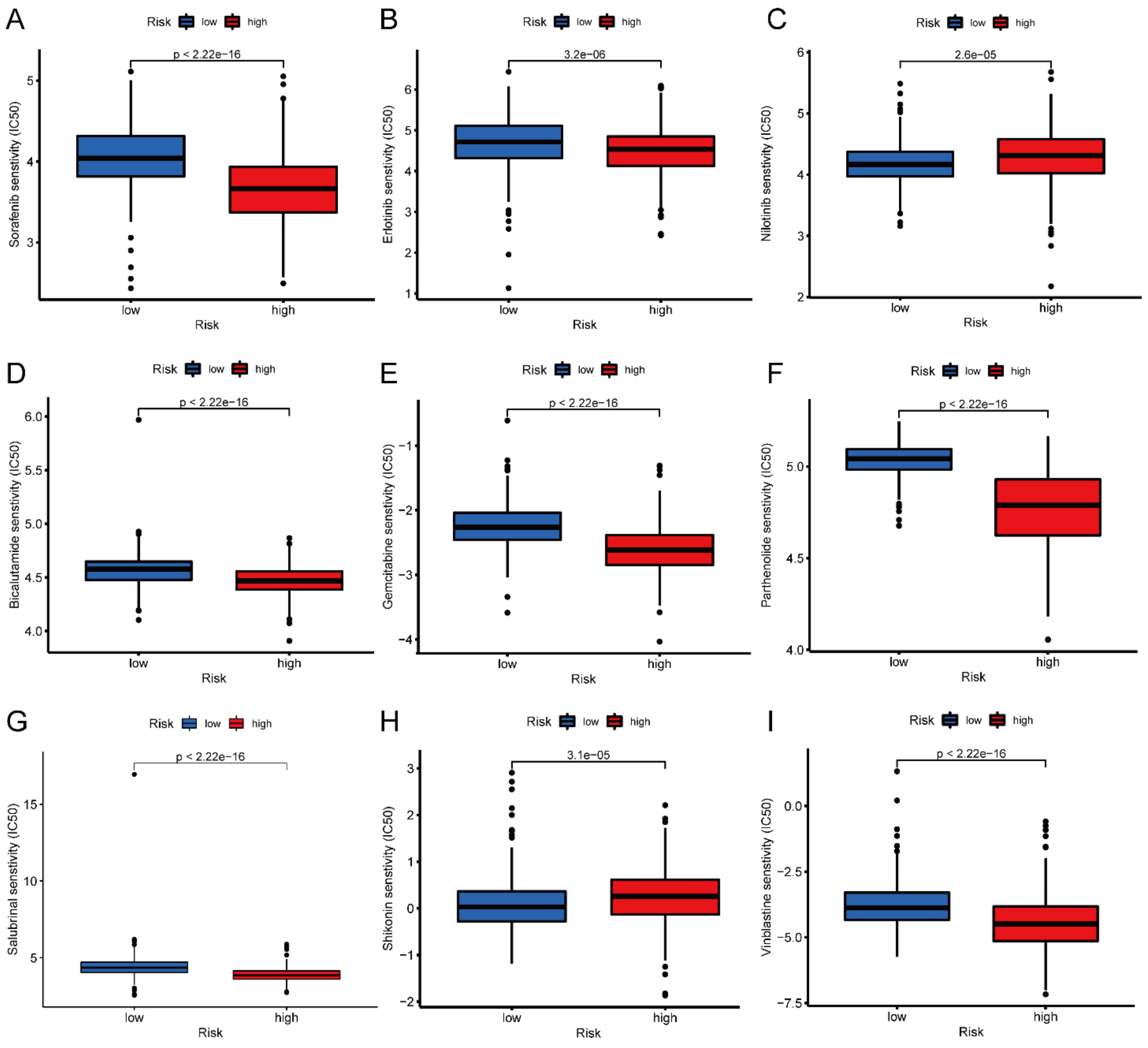

Supplementary Figure 4. Comparison of chemotherapy sensitivity in glioma between two risk groups.

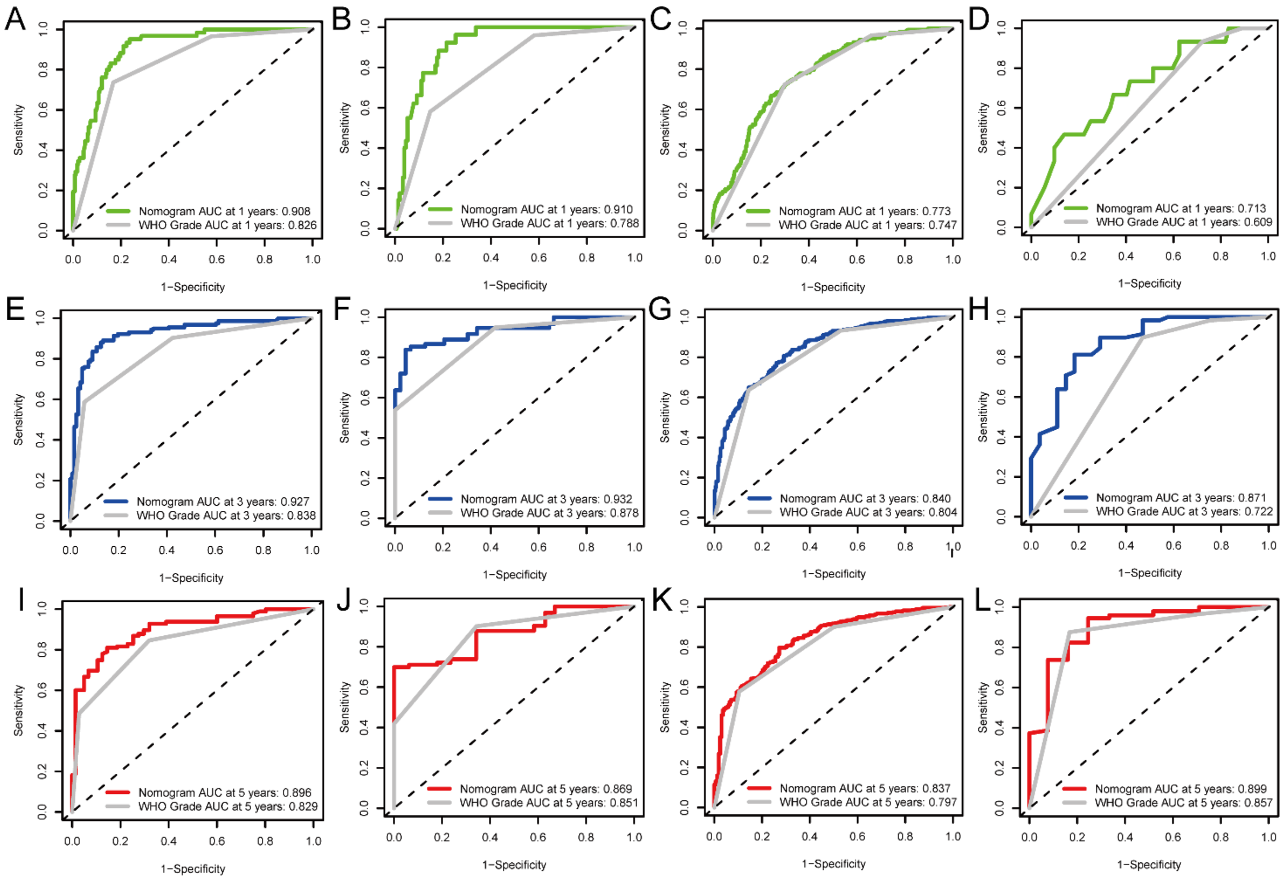

Supplementary Figure 5. Comparison of the nomogram and WHO grade in predicting the sensitivity and specificity of 1-year survival (A-D), 3-year survival (E-H), 5-year survival (I-L) in glioma patients from TCGA training dataset (A, E, I), TCGA testing dataset (B, F, J), CGGA (C, G, K) and Rembrandt (D, H, L).
